# L’aplasie médullaire acquise sévère et l’hépatite auto-immune séronégative: une association rare et grave

**DOI:** 10.11604/pamj.2019.34.132.20773

**Published:** 2019-04-23

**Authors:** Amine Benmoussa, Aznag Mohamed Amine, Rajaa Tissir, Illias Tazi

**Affiliations:** 1 Service d'Hématologie, CHU Mohammed VI, Marrakech, Maroc

**Keywords:** Pyoderma gangrenosum, dermatose neutrophilique, hyperleucocytose, Jaundice, pancytopenia, autoimmune hepatitis, marrow aplasia

## Abstract

Les aplasies médullaires post hépatitiques sont des aplasies sévères survenant dans les 6 mois suivant au moins un épisode d'hépatite clinique d'installation rapide généralement séronégative pour les virus d'hépatite connus. Nous rapportons le cas d'un patient de 28 ans présentant une hépatite auto-immune révélée par un ictère avec hépatomégalie et cytolyse hépatique et confirmée par une ponction biopsie hépatique; se compliquant 4 mois plutard par l'apparition d'une pancytopénie liée à une aplasie médullaire sévère.

## Abstract (en)

Post-hepatitis marrow aplasias are severe aplasias occurring in the six months following at least one episode of early onset hepatitis, usually seronegative for hepatitis viruses known. We report the case of a 28-year old patient with autoimmune hepatitis revealed by jaundice associated with hepatomegaly and hepatic cytolysis and confirmed by liver biopsy; four months later it was complicated by pancytopenia associated with severe marrow aplasia.

## Introduction

Les hépatites auto-immunes séronégatives représentent un groupe de maladies hétérogènes de cause inconnue, caractérisées par des lésions hépatocytaires nécrotico-inflammatoires avec la présence des anticorps particuliers et une grande corticosensibilité. Ce sont des pathologies rares pouvant affecter les 2 sexes de tout âge et se compliquent rarement d'aplasie médullaire sévère. Nous rapportons le cas d'un patient atteint d'hépatite auto-immune dont l'évolution est marquée 4 mois plus tard par la survenue d'une aplasie médullaire sévère [1, 2].

## Patient et observation

Il s'agit d'un patient âgé de 28 ans, sans antécédents pathologiques particuliers, qui présente depuis 2 semaines une douleur abdominale avec ictère, le tout évolue dans un contexte de conservation de l'état général. L'examen clinique initial trouve un patient conscient avec un score de Glasgow à 15/15^ème^, apyrétique T: 36,5°C, une tension artérielle à 12/7cmHg, une saturation en Oxygène à 100%, une fréquence cardiaque à 68 bat/min, un ictère généralisé, on note l'absence d'hépatomégalie et de splénomégalie, les aires ganglionnaires sont libres, le reste de l'examen somatique est normal. Le bilan biologique montre un hémogramme normal, une cytolyse L IJNhépatique avec une alanine amino-transférase (ALAT) à 900UI/L et une aspartate amino-transférase (ASAT) à 1100UI/L, une hyperbilirubinémie avec bilirubine conjuguée à 30 mg/l et bilirubine non conjuguée à 2 mg/l, une phosphatase alcaline normale, le bilan d'hémostase est normal, les sérologies (VIH, VHA, VHB, VHC, EBV, CMV) sont négatives, le bilan immunologique est négatif (Ac AntiDNA, Ac Antimucle lisse, Ac Antinucléaires, Ac Anti nucléocytoplasmique, Facteur rhumatoïde, Ac anticytosol hépatique). Le bilan radiologique montre une hépatomégalie à 16cm sans autres signes associés à l'échographie abdominale. Le bilan de la maladie de Wilson est négatif, la ponction-biopsie hépatique objective des lésions hépatocytaires nécrotico-inflammatoires. Le malade n'est pas mis sous aucun traitement du fait d'un retard de récupération des examens paracliniques sus cités, le tableau clinique s'aggrave après 4 mois de sa première consultation par l'apparition d'une asthénie profonde avec des lésions pétéchiales diffuses, l'hémogramme montre une anémie légèrement macrocytaire normochrome arégénérative avec Hb (hémoglobine) à 5g/dl, VGM (volume globulaire moyen) à 103 femtolitre, TCMH (Teneur corpusculaire en hémoglobine) à 28 et réticulocytes à 15 10^9^/L, un nombre de plaquettes à 4 10^9^/L, avec des globules blancs à 5 10^9^/L, des polynucléaires neutrophiles (PNN) à 1,3 10^9^/L, et des lymphocytes à 3,4 10^9^/L. le frottis sanguin montre la présence des macrocytes sans signes de dysplasie et sans cellules anormales. Le myélogramme est désertique, la biopsie ostéo -médullaire objective une moelle hypoplasique sans fibrose et sans présence des cellules tumorales, le clone HPN est négatif, le typage HLA montre une compatibilité du malade avec son frère. Le malade est mis sous ciclosporine (6mg/kg) en attendant la greffe des cellules souches hématopoïétiques, le bilan hépatique se normalise sans amélioration de bilan hématologique, le malade bénéficie par la suite d'une allogreffe des cellules souches hématopoïétiques mais l'évolution est défavorable avec décès du malade à J28 post greffe à cause de la maladie veino-occlusive.

## Discussion

Les aplasies médullaires (AM) acquises sévères sont des maladies rares, caractérisées par une atteinte secondaire du compartiment des cellules primitives médullaires. La destruction des cellules souches hématopoïétiques est probablement multifactorielle, associant une composante auto-immune et des altérations génétiques intrinsèques. L'AM est dite sévère si deux des critères suivants sont présents: polynucléaires neutrophiles < 0,5 10^9^/L, plaquettes < 20 10^9^/L, réticulocytes < 20 10^9^/L avec une moelle pauvre sur les explorations médullaires (cellularité médullaire < 25% ou comprise entre 25 et 50% avec moins de 30% de cellules hématopoïétiques résiduelles) [3, 4]. L'aplasie médullaire sévère associée à l'hépatite auto-immune est une forme bien décrite d'AM, survenant dans les 6 mois suivant une augmentation d'alanine aminotransférase (ALAT) d'au moins cinq fois de la limite supérieure des valeurs de référence, elle est généralement négative pour les virus hépatiques connus (A, B, C) [5]. Notre patient avait une hépatite auto-immune avec des chiffres de transaminases (ALAT, ASAT) > 5 fois la normale, avec des sérologies hépatiques normales, l'aplasie médullaire s'est installée 4 mois plus tard. L'hépatite auto-immune (HAI) séronégative est une affection rare, pouvant se compliquer d'aplasie médullaire dans 25% des cas. Lors de l'association de l'AM et l'HAI, cette dernière se caractérise par des épisodes d'ictère et de cytolyse hépatique, elle est d'évolution le plus souvent aigue, mais peut être parfois prolongée ou récurrente. Les anticorps spécifiques (Ac Antimucle lisse, Ac Antinucléaires, Ac anticytosol hépatique) de l'HAI sont généralement négatifs [6]. Notre patient avait une hépatite prolongée évoluant depuis 4 mois, et qui n'a pas été résolue qu'après administration de la ciclosporine, son bilan immunologique était négatif et le diagnostic de l'HAI a été retenu par ponction biopsie hépatique. Plusieurs études suggèrent que le principal mécanisme pathogène responsable de l'aplasie médullaire au cours de l'hépatite auto-immune est la dysrégulation immunologique du fait de la présence des anomalies immunologiques (HAI est caractérisée par un rapport diminué des cellules CD4/CD8 dans le sang périphérique, la présence des cellules T cytotoxiques activées secrétant interféron-gamma qui inhibe l'hématopoïèse) dans les HAI, et la bonne réponse observée après traitement immunosuppresseur [7, 8]. Dans le cas actuel l'hépatite auto-immune a régressé après traitement immunosuppresseur mais les signes d'aplasie ont persisté ce qui a imposé la réalisation de greffe des cellules souches hématopoïétiques. Le pronostic des aplasies médullaires sévères associées à des hépatites auto-immunes est défavorable et l'évolution est souvent fatale (ce sont des situations toujours mortelles en l'absence de traitement). Le traitement se base sur les soins de support visant la prise en charge des épisodes de la neutropénie fébrile par une antibiothérapie adaptée et la correction de l'anémie et la thrombopénie par des transfusions de culots globulaires en cas d'anémie profonde ou mal tolérée et plaquettaires en cas de syndrome hémorragique ou thrombopénie très profonde, et il repose aussi sur un traitement spécifique consiste à la greffe des cellules souches hématopoïétiques chez tout sujet jeune de moins de 40 ans avec donneur géno-identique (25% dans la fratrie) avec une survie plus de 80%, et sur le traitement immunosuppresseur à base de ciclosporine avec sérum anti lymphocytaire chez tout patient de plus de 40 ans ou sans donneur compatible avec une survie plus de 70%, mais avec risque d'évolution clonale vers une leucémie aigüe myéloïde ou myélodysplasie ou acquisition d'un clone HPN (hémoglobinurie paroxystique nocturne). Les patients qui ne répondent pas aux traitements immunosuppresseurs peuvent bénéficier d'une greffe de moelle osseuse non apparentée [9, 10]. Notre patient a été mis sous traitement symptomatique avec greffe de moelle osseuse.

## Conclusion

L'aplasie médullaire associée à l'hépatite auto immune est une situation très rare et sévère nécessitant un diagnostic rapide et une prise en charge immédiate et adaptée.

## Conflits d’intérêts

Les auteurs ne déclarent aucun conflit d'intérêts.
